# The humanistic care ability of nurses in 27 provinces in China: a multi-center cross-sectional study

**DOI:** 10.3389/fmed.2024.1450783

**Published:** 2024-08-19

**Authors:** Xiaoxiao He, Wei Wang, Lulu Liao, Yanhong Ren, Yilan Liu, Juan Xu

**Affiliations:** ^1^Department of Nursing, Union Hospital, Tongji Medical College Huazhong University of Science and Technology, Wuhan, Hubei, China; ^2^School of Nursing, Tongji Medical College Huazhong University of Science and Technology, Wuhan, Hubei, China; ^3^Health Science Center, Yangtze University, Jingzhou, Hubei, China; ^4^Department of Respiratory and Critical Care Unit, Union Hospital, Tongji Medical College Huazhong University of Science and Technology, Wuhan, Hubei, China

**Keywords:** Chinese nurses, 27 provinces, humanistic care ability, care, multi-center cross-sectional study

## Abstract

**Background:**

Currently, studies found that the humanistic care ability of nurses is at low level in China, resulting in patients’ concerns and dissatisfaction regarding the lack of empathy among nurses. We aimed to explore the factors that influence nurses’ humanistic care ability, providing a new perspective on improving patient satisfaction and promote high quality medical services.

**Methods:**

A multi-center cross-sectional study recruited nurses from tertiary and secondary hospitals in China between July 2022 and August 2022. Data concerning self-developed questions on nurses’ socio-demographic data and Caring Ability Inventory (CAI) were collected through the Questionnaire Star Platform, using a multi-stage sampling method.

**Results:**

The total score for the level of caring ability among the 15,653 surveyed Chinese nurses was 192.16 ± 24.94. Various factors significantly influence the level of humanistic care ability, including professional title, department, degree of passion for the job, job satisfaction, emphasis on self-care, participation in humanistic care training, support from family for the job, relationships with colleagues, satisfaction with salary, and previous experience working in pilot wards emphasizing humanistic care (*p* < 0.01).

**Conclusion:**

At present, nurses exhibit a comparatively modest proficiency in humanistic care ability. Numerous factors contribute to this situation. Nursing administrators ought to enhance the scope of humanistic care practices, conduct consistent professional training sessions, advocate for the implementation of model wards emphasizing humanistic care, foster a supportive organizational culture conducive to nurses, and underscore the significance of both nurturing nurses and promoting self-care among them.

## Introduction

Humanistic caring is the core of nursing (1), which advocates for patient-centered care, emphasizing the ability to meet patients’ individual requirements physiologically and psychologically, facilitate their comprehensive and unrestricted development and feel the value of life to develop therapeutic relationships (2). The World Health Organization highlights the vital importance of considering patients’ feelings and responsiveness in the evaluation framework of medical service systems (3). Nurses with advanced humanistic care ability, characterized by enhanced professional comprehensive quality and stronger service consciousness (4), are able to provide effective clinical practice and offer high quality of care, resulting in enhanced patient satisfaction and fostering a harmonious doctor-patient relationship (5).

The “Healthy China 2030” plan is a national strategy developed by the Chinese government aimed at improving the health of the general population, which includes enhancing healthcare workers’ humanistic care abilities to strengthen the provision of humanistic care in health services and ultimately improve patient satisfaction (6). Nevertheless, in hospitals with limited resources, there is a tendency to prioritize technology over humanistic values, which leads to nurses be accustomed to focus on disease treatment while disregarding activities that requires communication, encourage and accompany (7). Consequently, patients continue to express their concerns and dissatisfaction regarding the perceived lack of empathy among healthcare providers (8). Many research on humanistic care ability of nurses found that the total score was lower than standards (9–11). Therefore, there is a pressing need to prioritize and cultivate the humanistic care capabilities of nursing professionals.

Most research have explored factors of humanistic ability in nurses, socio-demographic data collected often used to analysis related characteristics. Age, gender, marital status, children status, education level, employment patterns, department and professional title have been observed to be influencing factors (10–15). Additionally, research has shown that psychological capital and self-efficacy positively affected the humanistic care ability of nurse (15), characteristics of solidified personality and little psychological resource result in a low level of humanistic care ability (16). Nurses with high levels of empathy (17), compassion (18), emotional quotient (EQ) (19) and communication ability (20) show a high level of work engagement which influence the realization of humanistic caring. Organizational climate (21) and social support (12) indicate a set of external characteristics plays an important role in nurses’ caring ability.

Training has the potential to improve the humanistic care abilities of nurses (22). Studies have shown that targeted training interventions can enhance humanistic care capabilities (2, 10), which is contingent on the internal properties of cognition, practice, and education, an integration of knowledge, attitude, emotion, and behavior (23).

This multi-center study aimed to explore the factors that influence nurses’ humanistic care ability, providing a new perspective on improving patient satisfaction and promote high quality medical services.

## Methods

### Design

This multicenter cross-sectional study enrolled nurses from tertiary and secondary hospitals in China between July 2022 and August 2022, using a multi-stage sampling method.

### Participants

**Figure 1:**
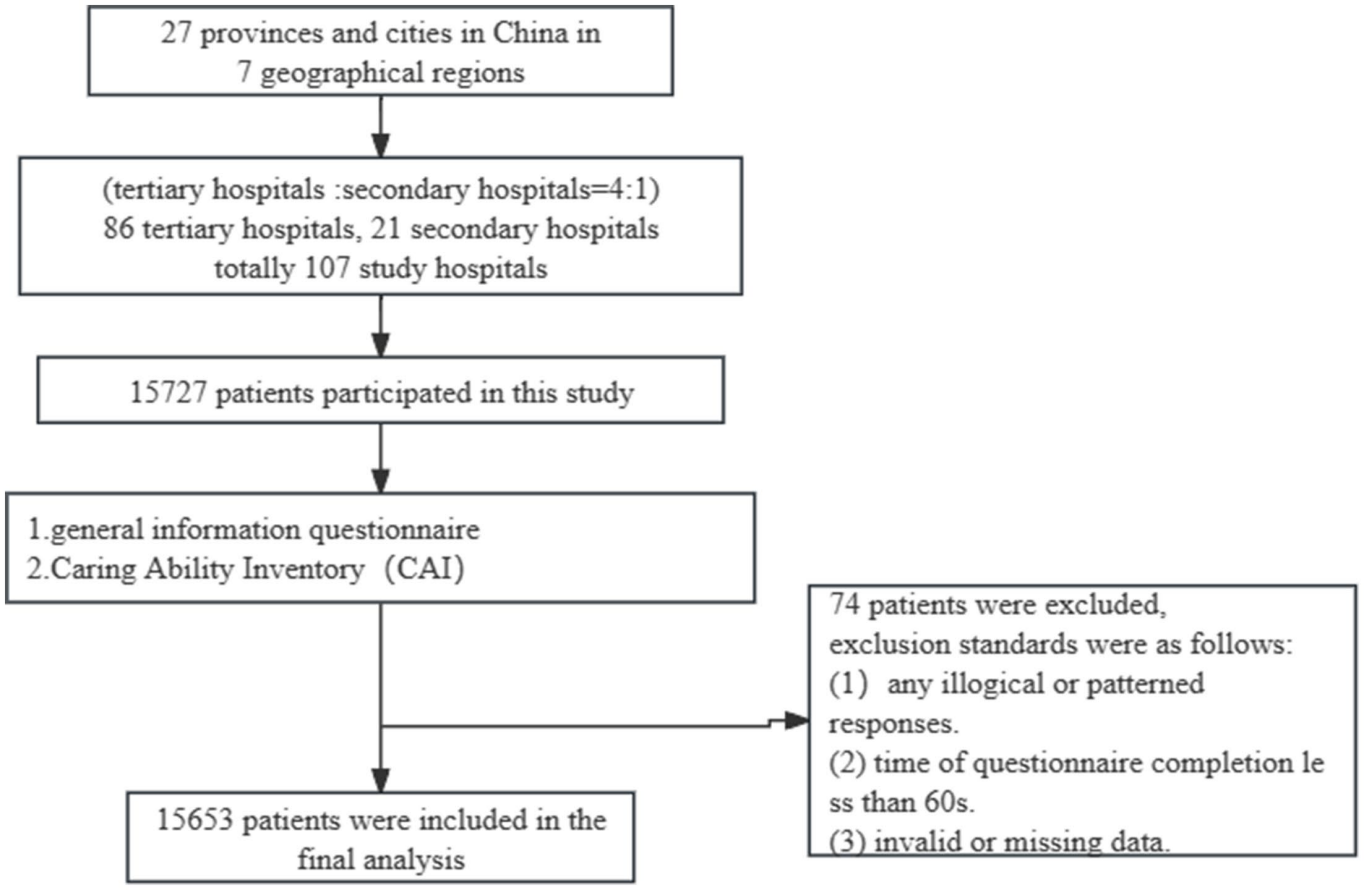
Flowchart of participants throughout the study.

The medical institutions surveyed were all members of the Humanistic Care Professional Committee of the Chinese Association for Life Care. Using a multi-stage sampling method for data collection from July 2022 to August 2022. During the first stage of the study, we selected 27 provinces and cities, 7 geographical regions according to the regional distribution of China Life Care Association’s hospital members. The sample size for each provincial unit was established based on its proportion to the total population of China. The sampling frame excluded Hong Kong, Macao, Taiwan. In the second stage, the number of tertiary and secondary hospitals to be included in the study was selected according to the radio of 4:1 after the approval of the nursing management of the hospital. At least 10 departments of nurses were selected in tertiary hospitals and 5 departments in secondary hospitals to ensure representation. Figure 1 presents a schematic diagram illustrating the process employed in this study.

The sample size was calculated by the following formula: *n* = *μ*^2^_*α*/2_*p*(1 − *p*)/*δ*^2^ (where *α* = 0.05, *μα*/2 = 1.96, *δ* = 0.05). *p* represents the involved nurses’ humanistic care ability (24), considering 20% non-response rates, the minimum sample size was 443. The inclusion criteria were as follows: ① registered nurses who have worked in clinical nursing for more than one year; ② worked continuously for at least one month prior to participating in this study; ③ nurses with informed consent. The exclusion criteria were as follows: nurses who are in further education, standardized training, internships, or on leave due to illness or maternity.

### Ethical consideration

Ethical approval was obtained from the Ethics Committee of Tongji Medical College, Huazhong University of Science and Technology, with an ethics approval number of 2022S161. This study was conducted in accordance with the Declaration of Helsinki as well as relevant guidelines and regulations (19). The developers permitted the use of CAI questionnaires. All nurses who volunteered to participate were required to sign a consent form after the aim and method of this study were explained to them.

### Instruments

The questionnaire included 2 parts: self-developed questions on nurses’ socio-demographic data and Caring Ability Inventory (CAI).

The socio-demographic survey sheet was designed including gender, age, education level, marital status, working years, professional title, duty, department, employment pattern, degree of familial support for work, relationship with colleagues, level of passion for work, degree of satisfaction with remuneration, level of job satisfaction, whether he/she had worked in a humanistic care pilot ward, extent of emphasis on self-care, whether he/she has teaching tasks, whether he/she had received training related to humanistic care and training method.

Caring Ability Inventory (CAI), developed by Nkongho (25) in 1990, is a tool used to test a person’s ability to care for others, and has been widely used in academy and clinic worldwide. It was translated into Chinese by Xu Juan in 2008 (16). The inventory was subsequently applied to 350 nursing staff in a tertiary hospital in Wuhan for reliability and validity tests. The scale is comprised of 37 items grouped into 3 dimensions of caring ability: cognition (14 items), courage (13 items), and patience (10 items). All items are graded using the Likert 7-level scoring method ranging from “totally disagree” to “totally agree,” with scores of 1–7 assigned to each response. The thirteenth item is reverse-scored. The total score ranges from 37 to 259 points, with higher scores indicating stronger humanistic care abilities among nursing staff. The Cronbach’s alpha was 0.84. International scoring standards suggest that scores below 203.1 indicate low ability, scores between 203.1 and 220.3 indicate moderate ability, and scores above 220.3 indicate high ability.

### Data collection

The survey was conducted using the Questionnaire Star Platform. The questionnaire included standardized instructions, and each item was designated as a mandatory response. Each IP address could only be submitted once. During the survey, approval for data collection was obtained from the directors of the participating hospitals that collected the data. A designated representative from each hospital was assigned to assist with the investigation. Following training conducted for all designated representatives by our research team, questionnaires were distributed within each department, ensuring that the nurses were informed and willing to participate before completing the survey, ensuring that the nurses were informed of the aim and significance of this study and were required to complete the questionnaire voluntarily. Our research team members provided timely feedback to the representatives on the response condition, ensuring that all participating hospitals completed the survey as required and on time. After the questionnaires were collected, two researchers cross-checked the data and the data exclusion standards were as follows: (1) any illogical or patterned responses. (2) Time of questionnaire completion less than 60 s. (3) Invalid or missing data.

### Data analysis

The original data exported from the Questionnaire Star platform were initially processed by Excel spreadsheet. According to the data exclusion standards, 15,653 valid data were reserved. SPSS 26.0 software were used for data analysis. Descriptive statistics such as frequency and percentage were used for categorical variables. For continuous variables following a normal distribution, the mean (*x*) and standard deviation (*s*) were used to describe the data. Independent samples *t*-test, analysis of variance (ANOVA) were employed for single-factor analysis in caring abilities. Independent variables (*p* < 0.05) in the univariate analysis were entered into stepwise multiple linear regression. Pearson correlation method was used for measuring the correlation between scales. The two-sided level was *α* = 0.05 and the overall statistically significance was *p* < 0.05.

## Results

### Demographic characteristics

The questionnaire received a total of 15,727 responses. After excluding invalid responses, 15,653 valid questionnaires were obtained, resulting in an effective response rate of 99.53%. The study included 27 provincial-level administrative regions, including Shanghai, Jiangsu, Anhui, Shandong, Fujian, Beijing, Tianjin, Shanxi, Hebei, Inner Mongolia, Henan, Hubei, Hunan, Guangdong, Guangxi Zhuang Autonomous Region, Hainan, Chongqing, Sichuan, Guizhou, Yunnan, Shaanxi, Gansu, Qinghai, Xinjiang Uygur Autonomous Region, Heilongjiang, Jilin, and Liaoning. A total of 107 hospitals were included in the survey, including 86 tertiary hospitals and 21 secondary hospitals. The survey included 15,653 participants aged 18 to 59 years (mean age 32.32 ± 6.65 years); with work experience ranging from 0 to 41 years, with a median of 9.00 years (interquartile range: 5.00 to 13.00 years) (see Tables 1, 2).

**Table 1:** Humanistic caring ability by participants’ characteristics and the single-factor analysis of the humanistic caring abilities of the surveyed subjects (*N* = 15,653).

Variables		*N*	Score $\left(\bar{x}\pm s\right)$	*t*/*F*	*p*-value
Hospital level	Tertiary hospitals	14,305	192.24 ± 24.72	1.126^a^	0.260
	Secondary hospital	1,348	191.37 ± 27.13		
Nature of the hospital	General hospital	13,454	192.37 ± 25.05	2.556^a^	0.011
	Specialized hospital	2,199	190.90 ± 24.21		
Gender	Male	690	191.46 ± 29.57	−0.645^a^	0.519
	Female	14,963	192.19 ± 24.71		
Department	Internal medicine	4,482	192.44 ± 25.45	4.211^b^	<0.001
	Surgical	3,558	193.02 ± 25.43		
	Obstetrics and gynecology	1,367	193.11 ± 24.02		
	Pediatric	950	191.76 ± 23.65		
	Outpatient/emergency department	922	189.05 ± 24.33		
	Operating room and ICU	2,284	189.50 ± 20.75		
	Other	2090	191.13 ± 23.60		
Education level	Secondary college	71	188.65 ± 27.60	1.579^b^	0.177
	Junior college	2,434	192.19 ± 24.42		
	Undergraduates	12,887	192.37 ± 27.72		
	Master degree and above	261	213.50 ± 30.41		
Professional title	Nurse	2,387	190.22 ± 28.32	8.338^b^	<0.001
	Nurse practitioner	7,274	191.75 ± 25.60		
	Nurse-in-charge	5,472	193.34 ± 22.94		
	Deputy chief physician	485	194.13 ± 16.87		
	Chief physician	35	197.37 ± 20.92		
Marital status	Unmarried	4,662	188.45 ± 25.81	50.477^b^	<0.001
	Married	10,706	193.67 ± 24.43		
	Widowed	285	196.41 ± 23.22		
Tittle	None	14,856	192.09 ± 25.24	−1.917^a^	0.056
	Nurse manager and above	797	193.41 ± 18.45		
Employment type	State-employed	2,751	195.03 ± 22.95	22.361^b^	<0.001
	Personnel agency	2,632	191.81 ± 23.72		
	Contract-employed	10,270	191.48 ± 25.69		
Relationships with colleagues	Very harmonious	9,200	196.75 ± 24.27	288.041^b^	<0.001
	Harmonious	5,650	186.54 ± 23.34		
	General	781	181.95 ± 29.64		
	Not harmonious	22	179.03 ± 47.64		
Family support for work	Very support	9,327	196.51 ± 24.33	267.470^b^	<0.001
	Support	5,151	186.87 ± 23.25		
	General	1,126	181.14 ± 27.81		
	Unsupportive	49	173.35 ± 42.60		
Passion for nursing	Very like	5,712	200.72 ± 25.21	495.260^b^	<0.001
	Like	6,458	190.26 ± 21.99		
	General	3,238	182.67 ± 24.20		
	Dislike	245	173.50 ± 31.60		
Satisfaction with salary	Very satisfied	2,786	203.80 ± 26.98	373.204^b^	<0.001
	Satisfied	5,834	193.54 ± 21.81		
	General	5,506	187.45 ± 24.04		
	Dissatisfied	1,527	182.62 ± 26.84		
Job satisfaction level	Very satisfied	3,768	203.29 ± 26.02	504.507^b^	<0.001
	Satisfied	7,056	192.28 ± 21.60		
	General	4,385	183.85 ± 24.48		
	Dissatisfied	444	178.01 ± 30.37		
Level of emphasis on self-care	Attach very importance to	5,753	200.34 ± 25.19	408.539^b^	<0.001
	Attach importance to	6,599	189.65 ± 22.23		
	General	3,064	183.00 ± 24.60		
	Attach no importance to	237	182.11 ± 33.46		
Ever worked in a pilot ward focused on humanities	Yes	4,468	194.46 ± 26.18	16.355^a^	<0.001
	No	11,185	190.04 ± 24.10		
To undertake teaching	Yes	7,033	270.39 ± 39.88	14.475^a^	<0.001
	No	8,620	260.98 ± 41.20		
Training on humanistic care	Yes	9,132 (58.3)	197.46 ± 26.18	13.331^a^	<0.001
	No	6,521 (41.7)	190.04 ± 24.10		

a *t*-value.

b *F*-value.

**Table 2:** Total scale and dimension scores of Nurses ‘Humanistic caring ability (*N* = 15,653).

Items	Items scores	Items total score	Level [*N* (%)]		
	(M ± SD)	(M ± SD)	Low	Medium	High
Knowing	5.67 ± 0.88	79.31 ± 12.37	6,289 (40.18)	3,116 (19.91)	6,248 (39.91)
Courage	4.04 ± 1.17	52.53 ± 15.17	12,160 (77.68)	2094 (13.38)	1,399 (8.94)
Patience	6.03 ± 0.75	60.32 ± 7.53	7,365 (47.05)	4,175 (26.67)	4,113 (26.28)
Total scale	5.19 ± 0.67	192.16 ± 24.94	11,695 (74.72)	2,217 (14.16)	1741 (11.12)

The overall scores and degree classification of the questionnaire on the humanistic caring abilities of the surveyed subjects still remain at a relatively low level, particularly in the dimension of courage.

### Multifactor analysis of the humanistic caring abilities of the surveyed subjects

In the multifactor analysis, statistically significant items from the univariate analysis were taken as independent variables (see Table 3 for independent variable assignments). The total score of humanistic caring abilities was considered as the dependent variable. Multiple linear regression analysis was conducted, and the results showed that 10 independent variables, including department and professional title, entered the regression equation (see Table 4).

**Table 3:** Assignment of independent variables.

Variable	Value
Nature of the hospital	0 = General hospital; 1 = Specialized hospital
Gender	0 = Male; 1 = Female
Professional title	1 = Nurse; 2 = Nurse practitioner; 3 = Nurse-in-charge; 4 = Deputy chief physician; 5 = Chief physician
Department	Internal medicine (Z1 = 0, Z2 = 0, Z3 = 0, Z4 = 0, Z5 = 0, Z6 = 0); surgical (Z1 = 1, Z2 = 0, Z3 = 0, Z4 = 0, Z5 = 0, Z6 = 0); obstetrics and Gynecology (Z1 = 0, Z2 = 1, Z3 = 0, Z4 = 0, Z5 = 0, Z6 = 0); pediatric (Z1 = 0, Z2 = 0, Z3 = 1, Z4 = 0, Z5 = 0, Z6 = 0); outpatient/emergency department (Z1 = 0, Z2 = 0, Z3 = 0, Z4 = 1, Z5 = 0, Z6 = 0); operating room and ICU (Z1 = 0, Z2 = 0, Z3 = 0, Z4 = 0, Z5 = 1, Z6 = 0); other (Z1 = 0, Z2 = 0, Z3 = 0, Z4 = 0, Z5 = 0, Z6 = 1)
Marital status	Unmarried (Z1 = 0, Z2 = 0); married (Z1 = 1, Z2 = 0); divorced or widowed (Z1 = 0, Z2 = 1)
Tittle	0 = None;1 = Nurse manager and above
Employment type	1 = State-employed; 2 = Personnel Agency; 3 = Contract-employed
Relationships with colleagues	1 = Very harmonious; 2 = harmonious; 3 = General; 4 = Not harmonious
Family support for work	1 = Very support; 2 = Support; 3 = General; 4 = Unsupportive
The passion for nursing	1 = Very like; 2 = Like; 3 = General; 4 = Dislike
Satisfaction with salary	1 = Very satisfied; 2 = Satisfied; 3 = General; 4 = Dissatisfied
Job Satisfaction Level	1 = Very satisfied; 2 = Satisfied; 3 = General; 4 = Dissatisfied
The level of emphasis on self-care	1 = Attach very importance to; 2 = Importance; 3 = General; 4 = Attach no importance to
Ever worked in a pilot ward focused on humanities	0 = Yes; No = 1
To undertake teaching	0 = Yes; No =1
Training on humanistic care	0 = Yes; No =1
Age	Original value
Working age	Original value

**Table 4:** Results of multivariate linear stepwise regression analysis of nurses’ humanistic caring abilities (*N* = 15,653).

Variables	*B*	SE	*β*	*t*	*p*-value	VIF
Constants	222.179	1.725	—	128.822	<0.001	—
Employment type	−0.695	0.267	−0.022	−2.607	<0.05	1.231
Professional title	−0.686	0.314	−0.021	−2.185	<0.05	1.636
Department (OR, ICU)	−0.148	0.055	−0.020	−2.702	<0.01	1.012
Job satisfaction level	−2.594	0.421	−0. 082	−6.161	<0.001	3.193
The level of emphasis on self-care	−3.630	0.293	−0.182	−12.375	<0.001	1.497
To undertake teaching	−1.076	0.419	−0.021	−2.567	<0.001	1.254
The passion for nursing	−3.047	0.371	−0.096	−6.161	<0.001	2.457
Relationships with colleagues	−2.612	0.386	−0.063	−6.768	<0.001	1.533
Satisfaction with salary	−1.233	0.317	−0.044	−3.885	<0.001	2.274
Ever worked in a pilot ward focused on humanities	−2.686	0.450	−0.049	−5.967	<0.001	1.204

*F* = 175.797, *p* < 0.001, *R*^2^ = 0.128, adjusted *R*^2^ = 0.127, Durbin–Watson’s = 1.984.

## Discussion

Given constraints in human resources, this study did not encompass all regions nationwide. Extensive research has been conducted on the topic of humanistic caregiving abilities. Nevertheless, conducting a large-scale single-sample multicenter study of this magnitude is unprecedented.

In this study, the overall caregiving ability score of Chinese nurses (192.16 ± 24.94) was significantly lower than the minimum standard set by Nkongho (14, 15, 26–28), consistent with previous research. Further analysis revealed significant differences in caregiving abilities across various dimensions, with cognition being the highest, followed by patience, and courage being the lowest. All three dimensions were below the standard level set by Nkongho. This disparity is closely intertwined with traditional Chinese culture and the existing education system (29). Traditional Chinese culture advocates expressing emotions in a restrained and subtle manner (30), leading to nurses being less proficient in expressing their care and lacking effective communication skills. This has further resulted in a lower level of humanistic care ability among Chinese nurses. With advancements in nursing education and research in China, a model of nursing education has been established, including undergraduate, master’s, and doctoral programs. Various forms of humanistic care training are being implemented, and the Chinese Association of Life Care is actively promoting the development of standards for humanistic care (31). All these measures have effectively increased awareness of the caring abilities of Chinese nurses. However, the tense doctor-patient relationship in China and the ongoing conflicts between nurses and patients have made clinical practitioners (32), including nurses, more cautious when dealing with a large number of patients (10), lacking the courage to express care. Good humanistic care abilities can promote harmony in nurse–patient relationships, improve patient compliance, and encourage active cooperation with treatment, thereby accelerating recovery and reducing hospitalization time. Therefore, nurses’ caring abilities directly impact the patient’s healthcare experience and satisfaction (33). It is recommended that managers increase the practical content of humanistic activities, actively foster nurses’ sense of identity, strengthen nurses’ caring beliefs, and implement humanistic concepts into nursing practices.

The study findings reveal that compared to lower-ranked nurses, those with higher professional titles exhibit greater maturity in caregiving abilities, owing to the experience and knowledge accumulated throughout their career development (27, 34). Higher-ranked nurses benefit from more opportunities for professional knowledge and skills training, enabling them to better cope with various diseases and nursing situations, and to understand individual differences and special needs of patients more comprehensively. Nursing managers should foster collaboration among nurses of different ranks, enhance training and guidance for lower-ranked nurses, and collectively provide comprehensive and high-quality nursing services to patients. Nurses in departments such as outpatient clinics, operating rooms, and ICUs demonstrate relatively lower levels of humanistic caregiving abilities. These departments operate in highly urgent work environments (35), requiring nurses to handle a large volume of patient visits, promptly address complex situations, and make rapid decisions (15), nurses in these departments tend to prioritize meeting patients’ physiological needs (36), often lacking awareness of holistic care. Nursing managers should recognize the differences in humanistic caregiving abilities among nurses in different departments and improve the nursing capabilities of specialized nurses through targeted intervention measures (37).

When nurses are satisfied with their work and compensation, and they have a strong passion for their profession, they are likely to be more engaged and committed to their roles (38). This increased engagement can lead to a greater investment of time and effort in humanistic care. Higher job satisfaction, compensation and passion often correlate with a more positive outlook on their profession and workplace. Nurses who are satisfied are more likely to approach their work with enthusiasm and a desire to make a meaningful difference in patients’ lives. Similarly, job satisfaction and passion can contribute to the development of empathy and compassion towards patients. Nurses who enjoy their work and feel fulfilled by it are more likely to empathize with patients’ experiences and provide compassionate care, which are key components of humanistic care (30, 39). High levels of job satisfaction and passion may also drive nurses to seek opportunities for professional growth and development. This could include attending training sessions or workshops focused on humanistic care, which can further enhance their abilities in this area. We can know that job satisfaction and passion for the profession create a conducive environment for nurses to cultivate and demonstrate higher levels of humanistic (40). Absolutely, this underscores the importance of nursing managers prioritizing the creation of a positive organizational environment conducive to fostering job satisfaction and passion among nurses. Such an environment not only benefits nurses but also directly correlates with enhanced patient outcomes and overall healthcare quality.

The study findings indicate that nurses with greater support from family, harmonious relationships with colleagues, and a high emphasis on self-care demonstrate higher levels of caregiving abilities. In the course of personal growth, the family significantly shapes an individual’s life perspective, values. Given the substantial stress in nursing roles, the understanding and support of family are crucial. Emotional support and encouragement from the family help nurses actively confront challenges in their work. Establishing positive relationships with colleagues contributes to creating a teamwork-oriented and supportive work environment. Within such an environment, nurses and colleagues can engage in effective information exchange and share experiences, collectively delivering high-quality nursing services to patients (41). Nurses who prioritize self-care effectively manage their emotions and stress, maintaining overall well-being. The provision of high-quality care depends on the nurse’s physical, mental, and emotional health (42). Nurses who value self-care not only have the energy to attend to the needs of patients but also communicate and interact with them patiently and empathetically (43). Enhancing nurses’ abilities in humanistic care requires not only support from family and society but also a conscious effort by nurses to prioritize self-care while implementing compassionate care for patients.

In Ma’s et al. (19) research, we can know that nurses who have received training in humanistic care exhibit significantly higher levels of humanistic care competence compared to those who have not undergone such training. Such training helps nurses establish emotional connections with patients, improve their communication skills (44), and enhance their coping abilities. Nurses who have undergone humanistic care training respect patients’ rights and dignity, actively respond to their needs, and maintain patience even when faced with pressure, providing service to patients. Nurses working in humanistic care pilot wards have more opportunities to practice and apply humanistic care skills as these wards emphasize not only providing psychological support to patients and promoting their involvement in decision-making but also focusing on their physical and mental well-being. Nurses engaged in teaching roles continuously improve their professional competence and humanistic care abilities by learning how to better impart nursing knowledge and skills to students (20). Teaching provides a platform for nurses to interact with students, share their experiences and deepen their understanding of humanistic care (45). Only by understanding the importance of humanistic care can nurses elevate their abilities in this area. Therefore, it is crucial for managers to conduct effective humanistic care training, actively promote the development of caring wards, and recognize the significance of nursing education in enhancing nurses’ humanistic care capabilities.

### Limitations of the study

However, this study also had many limitations. First, the study only included nurses from tertiary and secondary hospitals, neglecting those from primary hospitals. Second, the factors influencing humanistic care abilities were only considered from the perspectives of nurses’ individual and organizational levels, without addressing societal factors. Last, the survey was unable to cover all provinces in China, which may have resulted in biased findings. So longitudinal and quantitative studies are warranted to better understand the degree and duration of specific benefits.

## Conclusion

In conclusion, this study benefits from a large sample size and broad coverage. The multi-center survey revealed that overall, nurses’ abilities in humanistic care remains suboptimal. Nurses with higher professional titles, a focus on self-care, prior training in humanistic care, strong family support for their work, good relationships with colleagues, passion for their profession, satisfaction with their work and compensation, and those who have worked in humanistic care pilot wards tend to have higher levels of humanistic care proficiency. However, nurses in operating rooms, ICUs, and emergency departments need improvement in their humanistic care abilities. These findings provide nursing managers with new perspectives for management. In future clinical management, nursing managers should focus more on enriching humanistic care practices, conducting regular professional training in humanistic care ability, promoting the development of humanistic care demonstration wards, fostering a positive organizational atmosphere to provide support for nurses, and emphasizing both care for nurses and their self-care. Enhancing nurses’ humanistic caregiving abilities is essential for improving the quality of nursing and patient satisfaction.

## Data Availability

The original contributions presented in the study are included in the article/supplementary material, further inquiries can be directed to the corresponding authors.
